# Does testosterone influence the association between sleep and frailty in men: results from the European Male Aging Study

**DOI:** 10.1186/s12877-023-04450-8

**Published:** 2023-12-06

**Authors:** Seema D. Sharma, Michael J. Cook, Leen Antonio, Evelien Gielen, Gyorgy Bartfai, Felipe F. Casanueva, Ilpo T. Huhtaniemi, Mario Maggi, Margus Punab, Giulia Rastrelli, Jolanta Slowikowska-Hilczer, Jos Tournoy, Dirk Vanderschueren, Frederick C. Wu, Terence W. O’Neill

**Affiliations:** 1 Centre for Epidemiology Versus Arthritis, Division of Musculoskeletal and Dermatological Sciences, Faculty of Biology Medicine and Health, University of Manchester, The Stopford Building, Oxford Road, Manchester, M13 9PT UK; 2 Department of Clinical and Experimental Medicine, KU Leuven, Laboratory of Clinical and Experimental Endocrinology, Louvain, Belgium; 3 Department of Endocrinology, University Hospitals Leuven, Louvain, Belgium; 4 Centre for Metabolic Bone Diseases, Department of Geriatrics, University Hospitals Leuven, Louvain, Belgium; 5 Department of Obstetrics, Gynaecology and Andrology, Albert Szent-Gyorgy Medical University, Szeged, Hungary; 6 Department of Medicine, CIBER de Fisiopatología Obesidad y Nutricion, Santiago de Compostela University, Complejo Hospitalario Universitario de Santiago (CHUS), Instituto Salud Carlos III, CB06/03 Santiago de Compostela, Spain; 7 Institute of Reproductive and Developmental, Department of Metabolism, Digestion and Reproduction, Imperial College London, Hammersmith Campus, London, UK; 8 Andrology Unit, Mario Serio Department of Experimental and Clinical Biomedical Sciences, University of Florence, Florence, Italy; 9 Andrology Clinic, Tartu University Hospital, Tartu, Estonia; 10 Institute of Clinical Medicine, University of Tartu, Tartu, Estonia; 11 Institute of Biomedicine and Translational Medicine, University of Tartu, Tartu, Estonia; 12 Department of Andrology and Reproductive Endocrinology, Medical University of Łódź, Łódź, Poland; 13 Department of Geriatrics, University Hospitals Leuven, Louvain, Belgium; 14 Department of Public Health and Primary Care, KU Leuven, Louvain, Belgium; 15 Department of Endocrinology, Manchester University NHS Foundation Trust, Manchester, UK; 16 NIHR Manchester Biomedical Research Centre, Manchester Academic Health Science Centre, Manchester University NHS Foundation Trust, Manchester, UK

**Keywords:** Sleep, Frailty, Ageing, Testosterone, EMAS study

## Abstract

**Background:**

Previous studies have suggested an association between sleep disturbance and frailty. The mechanism is unknown, although it has been suggested that hormonal factors may play a role.

**Methods:**

The aim was to determine the association between sleep duration, sleep quality and frailty, and to determine whether testosterone influenced this association. Males aged 40–79 years were recruited from eight European centres to the European Male Aging Study (EMAS). Subjects completed an interviewer-assisted questionnaire including questions regarding sleep quality and duration. Sleep quality was scored 0–20 and categorised as 0–4, 5–9, 10–14, and 15–20, with higher scores indicating poorer quality. A 39-component frailty index (FI) was constructed. Total testosterone levels were measured. The association between sleep duration, sleep quality and the FI was assessed using negative binomial regression, with adjustment for putative confounders including testosterone level.

**Results:**

Two thousand three hundred ninety-three participants contributed data to the analysis. The mean age was 63.3 years and mean sleep duration was 7.01 h. The mean frailty index was 0.15. Mean testosterone levels declined with decreasing sleep quality. After adjustment, compared to those with a sleep score of 0–4, the FI was 57% (95% CI 38%, 78%) higher among those with a sleep score of 15–20. After adjustment compared to those with normal sleep duration *(6–9 h)*, those with a short *(< 6 h)* and long *(≥ 9 h)* sleep duration had a 16% (95% CI 6%, 28%) and 11% (95% CI 0%, 23%) higher FI, respectively. Adjustment for testosterone did not influence the strength of either association.

**Conclusion:**

Frailty is associated with impaired sleep quality and sleep duration. The association cannot, however, be explained by variation in testosterone levels.

**Supplementary Information:**

The online version contains supplementary material available at 10.1186/s12877-023-04450-8.

## Introduction

Frailty is a clinical state characterized by age-related deregulation of multiple physiologic systems, resulting in a loss of homeostatic capacity, and an increased risk of morbidity, disability, and death [1]. Adverse outcomes associated with frailty include falls, fractures, functional decline and admissions to nursing homes [2, 3].

Several studies have suggested a link between sleep disturbance and frailty [4–11]. Previous cross-sectional studies suggest an association between frailty and sleep disturbance. These include assessment of sleep by both self-reported and objective measures of assessment, such as actigraphy and formal sleep studies [4–11]. Those with sleep disturbance are more likely in prospective analysis to develop frailty [6].

Most studies suggest also a link between increased sleep duration and frailty, while some report an association between low sleep duration and frailty [4, 12–17].

The mechanism linking sleep and frailty is unknown but is likely to be multifactorial. It has been suggested that perturbations in biochemical pathways including alterations in testosterone levels may play a role. In multiple studies, low testosterone has been associated with frailty, including the European Male Aging Study (EMAS). These are summarised in recent systematic review and meta-analysis [18, 19]. Numerous studies investigating the relationship between testosterone levels and sleep parameters have shown an association between low levels of testosterone and impaired sleep [20–24]. To our knowledge, no studies have looked at the impact of testosterone on the relationship between sleep and frailty.

Using data from EMAS, we looked at the association between sleep quality, sleep duration and frailty in middle age and older men. We looked also at whether the association could be explained by variations in sex hormone levels.

## Methods

### Study design

Men aged 40–79 years were recruited for participation in the European Male Aging Study (EMAS), from population registers in eight centres: Florence (Italy), Leuven (Belgium), Łódź (Poland), Malmö (Sweden), Manchester (UK), Santiago de Compostela (Spain), Szeged (Hungary) and Tartu (Estonia). Details regarding recruitment and study set-up have been described in detail elsewhere [25]. In brief, subjects were invited for a postal questionnaire and then to attend for assessment at a local health / research facility. This comprised of an interviewer-assisted questionnaire, functional performance measures (outlined below) and a fasting blood sample. Subjects were invited to attend follow-up assessments after a mean of 4.3 years. Of those recruited at baseline, 86% (corrected for mortality) of patients completed follow up assessments. Questionnaire data included in analysis were obtained as part of the follow-up assessment, as information on sleep parameters were only collected in the follow-up assessment. Ethical approval for the study was obtained in accordance with local institutional requirements in each centre. Written, informed consent was obtained from all participants [25].

### European Male Ageing Study (EMAS): assessments

Participants all initially completed a postal questionnaire which comprised of demographic and lifestyle questions including the presence of chronic conditions, smoking status and alcohol consumption.

Subjects completed in-person interviewer-assisted questionnaires and assessments which included: the Becks Depression Inventory (BDI) [26]; the Physical Activity Scale for the Elderly [27]; the short form SF-36 questionnaire which was used to assess quality of life [28]; Reuben’s Physical performance test [29], the Adverse Life Events Scale [30]; the International Prostate Symptom score to assess lower urinary tract symptoms [31]; the Tinetti test for balance and postural stability [32]. Three validated neuropsychological tests were conducted to evaluate cognitive function; these measured visuo-perceptual abilities, executive function and memory [33], the recognition component of visual memory retrieval [34] and cognitive processing speed and visual scanning [35]. Information on pain was also collected by questionnaire and “chronic widespread pain” was classified by the American College of Rheumatology definition. Patients who reported pain but did not satisfy these criteria were classified as having “some pain” [36]. These assessments were used in the construction of the FI (see Supplementary Table 1). Height and weight were assessed and body mass index (BMI) was determined by dividing body weight in kilograms by height (m) squared.

### Sleep

At the follow-up survey information on sleep quality and sleep duration was collected using a questionnaire adapted from the Air Traffic Controller Health Change Study (the Jenkins Sleep Questionnaire) [37]. This is a well validated 4-item tool where participants were asked to consider on how many days of the month they; i) had difficulty falling asleep, ii) woke up several times a night, iii) had difficulty staying asleep including waking up far too early and, iv) how often they woke up after a normal amount of sleep feeling tired and worn out. Each question had six possible responses (Response: none, 1–3 days, 4–7 days, 8–14 days, 15–21 days, 22–31 days). Values were totalled to produce a score which ranged from 0 – 20, with higher scores indicative of worse sleep quality. We categorised sleep score into four groups; 0–4, 5–9, 10–14 and 15–20. Categorisation into distinct groups was performed to try and illustrate clinically meaningful differences in sleep score that may have distinct impacts on frailty, which may help suggest if there is a threshold effect. This may therefore allow for consideration of where one may target intervention.

Participants were additionally asked during the past month, on average how many hours sleep they had each night (see Supplementary Table 2). Sleep duration was categorised into three groups; normal; defined as between 6 and 9 h; short duration—defined as < 6 h and long – defined as ≥ 9 h.

### Assessment of frailty

The EMAS FI was constructed using a total of thirty-nine health deficits, all of which met criteria for use in a FI [38]. These included a range of self-reported comorbidities, selected items from the BDI-II and SF-36, impaired physical function as well as low cognition (see Supplementary Table 1). Continuous variables were dichotomised at the value of the 10th centile. Binary variables were characterised as “0” which represented absence or “1” which represented the presence of a deficit. For categorical variables, a value of “0.5” was used for an intermediate response. The total number of health deficits present in each participant was divided by the total 39 deficits that were considered, to give a measurement from 0–1. We also categorised the FI as “robust” (FI ≤ 0.2), 0.2 – 0.35 as “pre-frail” (0.2 < FI ≤ 0.35) and “frail” (FI > 0.35), based on previously published data [39].

### Testosterone

Subjects had a single fasting morning venous blood sample taken at baseline assessment. Total testosterone was measured using gas chromatography – mass spectrometry (GC–MS) in nmol/L [40]. A “low” total testosterone was defined as below 10.5 nmol/L and “normal” was considered to be 10.5 nmol/L or above, which reflects the median value of T thresholds for hypogonadal symptoms [41]. In order to derive free Testosterone levels, Vermeulen’s formula was used incorporating total T, SHBG and albumin concentrations [42].

### Statistical analysis

Descriptive statistics were used to describe the subject characteristics with continuous variables expressed as means and standard deviations, and dichotomous variables as counts and percentages. Box plots were used to show the association between sleep duration, sleep quality, and frailty. We looked at mean testosterone levels, and also the proportion of subjects defined as having a low testosterone by categories of sleep quality and sleep duration. Using linear regression we looked at the association between mean testosterone (outcome, continuous variable), categories of sleep quality and sleep duration with models adjusted for age and centre, and fully adjusted models adjusted for age, centre, BMI, depression, pain, smoking status and alcohol intake. Using logistic regression we looked also at the association between low testosterone (< 10.5 nmol) and sleep quality/duration categories, with the results expressed as odds ratios (OR) and 95% Confidence Intervals (CI).

We used negative binomial regression to look at the association between frailty index as a continuous variable, and both the quality of sleep and sleep duration categories. Negative binomial regression is a method used to model over-dispersed count variables. To facilitate this approach, FI was first multiplied by 100. 0 equated to no deficits and 100 equated to maximum possible deficits. In the analyses we looked separately at the associations between frailty and sleep duration and sleep quality with models adjusted initially for age and centre (model 1), followed by adjustment for BMI, pain, depression, alcohol and smoking (model 2) and a model further adjusted for testosterone (model 3). In a final model we included both sleep duration and sleep quality (model 4).

We repeated the analysis after categorising the FI (robust, pre-frail, and frail). In these analyses we used multinomial regression with the ‘robust’ group as the reference category. Results are reported as relative risk ratios (RRR) in the multinomial regression analysis, all with 95%CI. STATA/IC 14.0 (StataCorp, college Station, TX, USA) was used to perform all analysis.

## Results

### Participants

A total of 2,736 men participated in the EMAS follow up study. 2,393 men had complete data on frailty status, sleep quality and sleep duration and contributed to the analysis. Subject characteristics are summarised in Table 1. The mean age of the participants was 63.3 years (SD 10.5) and mean BMI 27.8 kg/m^2^. Mean frailty index (FI) was 0.15 (SD 0.12). 1,814 (75.8%) participants were robust, 410 (17.1%) were pre-frail and 169 (7.1%) were frail. The mean total testosterone was 16.4 nmol/L. 19.4% of participants had evidence of depression (as determined by BDI score of 11 or over), 16.5% were current smokers, 30.6% drank alcohol on 5 or more days a week and 8.8% reported chronic pain.

**Table 1:** Subject Characteristics

Variable	Mean	(SD)
Age (years)	63.3	(10.5)
Body mass index (kg/m^2^)	27.8	(4.3)
Sleep duration (hours)	7.01	(1.16)
Total T (nmol/L)	16.4	(6.2)
Frailty (frailty index)	0.15	(0.12)
	N	(%)
Depression		
BDI^1^ < 10	1905	(79.6)
BDI^1^ > 11	465	(19.4)
Frequency of alcohol consumption		
< 5 days a week	1,368	(57.2)
> 5 days a week	732	(30.6)
Smoking		
Non-smoker	1,961	(82.0)
Current smoker	395	(16.5)
Pain		
No pain	917	(38.3)
Some pain	1121	(46.8)
Chronic widespread pain	211	(8.8)
Sleep score		
0 – 4	1372	(57.3)
5 – 9	664	(27.8)
10 – 14	256	(10.7)
15 – 20	101	(4.2)
Sleep duration		
Short (< 6 h)	204	(8.5)
Normal (≥ 6 & < 9 h)	2027	(84.7)
Long (≥ 9 h)	162	(6.8)
Testosterone		
(> 10.5 nmol/L)	2006	(83.8)
(≤ 10.5 nmol/L)	345	(14.4)

^1^Beck’s depression inventory

Missing data: BMI = 46, depression = 23, alcohol = 293, smoking = 37, pain = 144, testosterone = 42

### Sleep quality and sleep duration

Just over half of participants (57.3%) had a sleep quality score between 0 and 4. 27.8% had a score between 5 and 9, while 4.2% had a score between 15 and 20. Mean sleep duration was 7.01 h. Most men (n = 2027, 84.7%) slept for between 6 and < 9 h. 162 (6.8%) and 204 (8.5%) had a longer and shorter sleep duration respectively, see Table 1.

### Sleep and testosterone

There was a small though non-significant decrease in mean serum total testosterone with increasing sleep score (see Table 2). The non-significant difference persisted after adjustment for age and centre, BMI, depression, pain, smoking and alcohol intake. The proportion of participants with low total testosterone (< 10.5 nmol/l) increased with increasing sleep score; from 13.5% among those with a low score (0–4) to 19.5% among those with a high score (15–20). After adjustment for covariates compared to those with a low score (0–4) those with a high score (15–20) were significantly more likely to have a low total testosterone level, (OR = 2.22; 95% CI 1.16, 4.31) (see Table 2). Those with higher levels of sleep disturbance also had lower levels of free testosterone, and this trended towards significance (see Supplementary Table 3).

**Table 2:** Association between testosterone, sleep quality score and sleep duration

Sleep Variable	Total Testosterone (nmol/L)	*Model 1*	*Model 2*	Low Testosterone (< 10.5 nmol/L)	*Model 3*	*Model 4*
*Sleep quality category*	*Mean (SD)*	β [nmol/L change in testosterone] (95% CI)		*N (%)*	Odds ratio for low testosterone (95% CI)	
0–4	16.6 (6.0)	**Reference**	**Reference**	183 (13.5%)	**Reference**	**Reference**
5–9	16.2 (6.4)	-0.41 (-1.00, 0.17)	-0.18 (-0.81, 0.45)	98 (15.1%)	1.11 (0.85, 1.46)	1.11 (0.80, 1.55)
10–14	15.9 (6.5)	-0.85 (-1.69, -0.02)*	-0.39 (-1.32, 0.54)	45 (17.9%)	1.42 (0.99, 2.05)	1.47 (0.92, 2.34)
15–20	15.9 (7.3)	-0.52 (-1.81, 0.76)	-0.68 (-2.08, 0.73)	19 (19.5%	1.35 (0.79, 2.31)	2.22 (1.16, 4.31)*
*Sleep duration*						
≥ 6 & < 9 h	16.5 (6.3)	**Reference**	**Reference**	284 (14.2%)	**Reference**	**Reference**
< *6 h*	15.8 (5.9)	-0.37 (-1.27, 0.54)	-0.05 (-1.02, 0.91)	31 (15.6%)	1.01 (0.67, 1.52)	1.04 (0.64, 1.69)
≥ 9 h	15.6 (6.3)	-0.94 (-1.96), 0.08)	-0.98 (-2.06, 0.09)	30 (19.6%)	1.42 (0.92, 2.17)	1.37 (0.82, 2.29)

Models 1 and 2 are constructed using linear regression. Models 3 and 4 are constructed using logistic regression. Model 1: Adjusted for age and centre, n = 2351; Model 2: adjusted for age, centre, BMI, depression, pain, smoking status and alcohol intake, n = 1885; Model 3: Adjusted for age and centre, n = 2351; Model 4: Adjusted for age, centre, BMI, depression, pain, smoking status and alcohol intake, n = 1885

^*^p < 0.05

Mean total testosterone was slightly higher in those with normal sleep duration (6–9 h) than those with longer or shorter mean sleep duration (15.6 nmol/l and 15.8 nmol / vs 16.5 nmol / l respectively) though the differences were not statistically significant. Compared to those with sleep duration of between 6 and 9 h the proportion of participants with low total testosterone (< 10.5nmol/l) was slightly greater in those with both a longer or shorter sleep duration (19.6% and 15.6% vs 14.2% respectively) though again the differences were not statistically different (Table 2).

### Sleep and frailty index

**Fig. 1:**
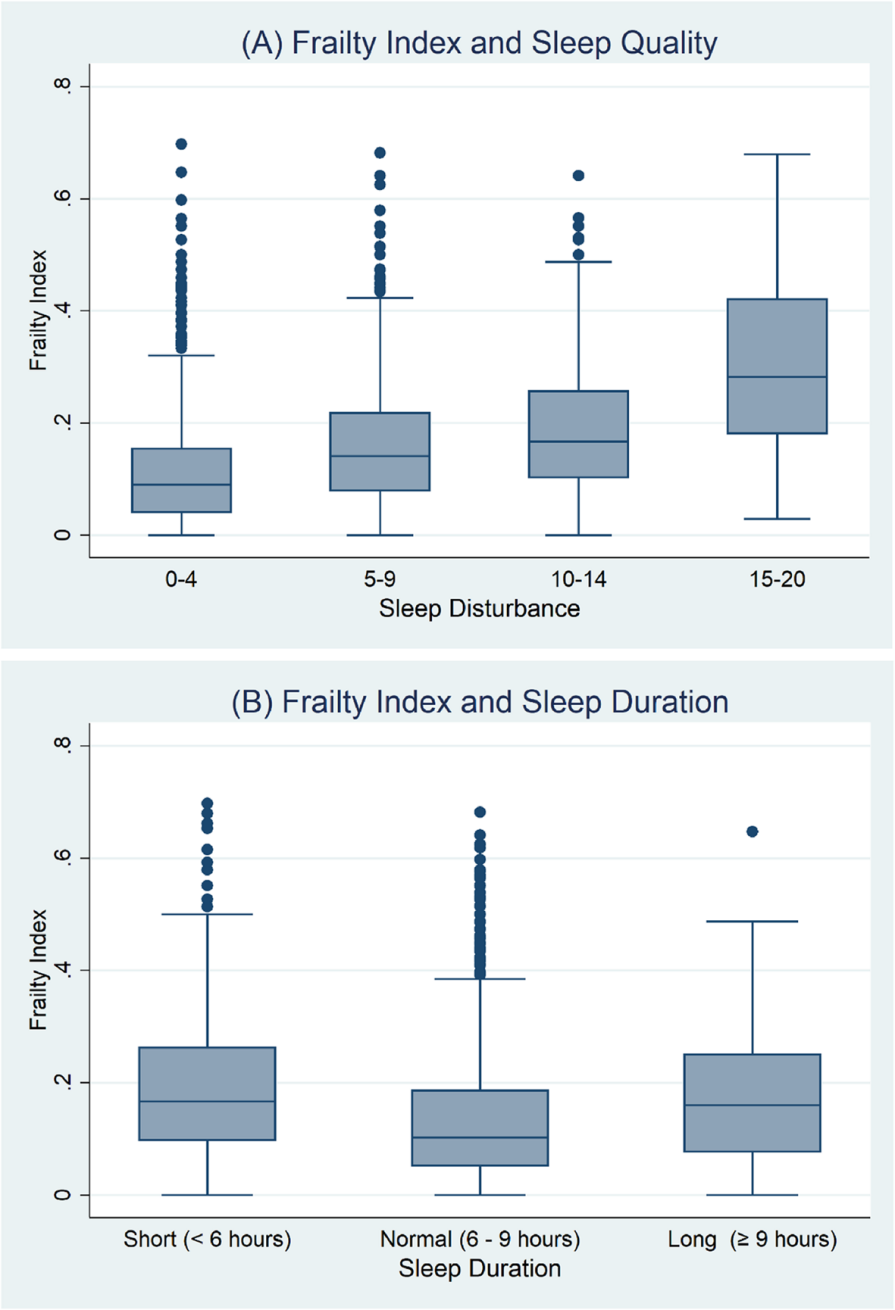
Box plots (**A**) Frailty Index by Sleep Quality Score (**B**) Frailty Index by Sleep Duration

FI increased with decreasing sleep quality (high sleep scores are indicative of poor sleep), see Fig. 1A. This remained true after adjustment for age and centre (model 1), and further adjustment for BMI, pain, depression, alcohol and smoking (model 2), (see Table 3). Compared to those with a sleep score of 0–4, those with score of 15–20 had a higher frailty index (relative FI = 1.57; 95% CI 1.38, 1.78). Further adjustment for a low total testosterone did not influence the strength of the association. The effect appeared independent also of sleep duration (model 4). Results were similar when using free testosterone instead of low total testosterone (see Supplementary Table 4).

**Table 3:** Association between sleep quality score, sleep duration and the frailty index

Sleep Variable	Model 1	Model 2	Model 3	Model 4
Relative FI score (95% CI)				
*Sleep quality category*				
0–4	**Reference**	**Reference**	**Reference**	**Reference**
5–9	1.40 (1.32, 1.49)**	1.26 (1.18, 1.34)**	1.26 (1.19, 1.34)**	1.27 (1.20, 1.35)**
10–14	1.80 (1.65, 1.96)**	1.37 (1.25, 1.49)**	1.37 (1.25, 1.49)**	1.38 (1.26, 1.51)**
15–20	2.47 (2.17, 2.81)**	1.57 (1.38, 1.78)**	1.52 (1.33, 1.73)**	1.53 (1.34, 1.75)**
*Sleep duration*				
≥ 6 & < 9 h	**Reference**	**Reference**	**Reference**	**Reference**
< *6 h*	1.43 (1.30, 1.58)**	1.16 (1.06, 1.28)**	1.14 (1.04, 1.25)*	1.01 (0.92, 1.11)
≥ 9 h	1.13 (1.01, 1.27)*	1.11 (1.00, 1.23)*	1.12 (1.01, 1.24)*	1.18 (1.07, 1.31)*

All models are constructed using negative binomial regression analysis. Model 1: Adjusted for age, centre, *n* = 2393; Model 2: Adjusted for age, centre, BMI, pain, depression, alcohol and smoking, *n* = 1903; Model 3: Same as model 2 but additionally adjusted for total testosterone, *n* = 1885; Model 4: Same as model 3 but mutually adjusted for both sleep quality and sleep duration, *n* = 1885

^**^P < 0.001

^*^P < 0.05)

Compared to those with normal sleep duration, FI was higher among those with a sleep duration of less than 6 h and ≥ 9 h, see Fig. 1B. This remained true after adjusting for age and centre (model 1) and further adjustment for BMI, pain, depression, alcohol and smoking (model 2) (Table 3). Compared to those with sleep between 6 and < 9 h, those with less than 6 h had a higher FI (relative FI = 1.16 (95% CI, 1.06, 1.28) while those with a longer sleep duration (9 h or more) had an increased FI also (relative FI = 1.11 (95% CI 1.00, 1.23)). Further adjustment for low total testosterone did not influence the strength of the relationship. The effect of longer (though not shorter) sleep duration on FI appeared to be independent of sleep quality (model 4).

### Sleep and frailty status

We looked also at the associations between sleep and frailty after categorisation of the FI (robust, prefrail and frail). In multinomial regression analysis, after adjustment for age, centre, BMI, pain, depression, alcohol intake and low total testosterone, compared to those with score 0–4, those with a low quality sleep (15–20) were more likely to be frail (RR = 9.88 (95% CI, 4.22, 23.14) and prefrail (RR = 3.34 (95% CI 1.69, 6.62) (model 3) (see Table 4). Compared to those who slept 6–8 h per day those who slept longer (RR = 1.32) and shorter (RR = 1.84) were more likely to be frail though the confidence intervals around these estimates embraced unity (see Table 4). As with frailty index, the effect of longer (though not shorter) sleep duration appeared to be independent of sleep quality (model 4) (see Table 4). Results were again similar when using free testosterone instead of low testosterone (see Supplementary Table 5).

**Table 4:** Association between sleep quality score, sleep duration and the frailty index category

Sleep Variable	Model 1		Model 2		Model 3		Model 4	
Relative FI score (95% CI)								
	**Pre-frail**	**Frail**	**Pre-frail**	**Frail**	**Pre-frail**	**Frail**	**Pre-frail**	**Frail**
Sleep quality								
0–4	**Reference**	**Reference**	**Reference**	**Reference**	**Reference**	**Reference**	**Reference**	**Reference**
5–9	2.30 (1.76, 3.03)**	2.93 (1.88, 4.56)**	1.89 (1.37, 2.61)**	1.91 (1.09, 3.34)*	1.96 (1.41, 2.71)**	2.05 (1.17, 3.61)*	2.04 (1.47, 2.84)**	2.12 (1.20, 3.74)*
10–14	4.17 (2.91, 5.99)**	6.82 (3.97, 11.72)**	2.58 (1.66, 4.02)**	2.72 (1.35, 5.48)*	2.64 (1.69, 4.13)**	2.74 (1.34, 5.61)*	2.91 (1.83, 4.61)**	2.96 (1.43, 6.16)*
15–20	7.89 (4.41, 14.11)**	41.90 (21.93, 80.03)**	3.39 (1.71, 6.71)**	10.93 (4.76, 25.14)**	3.34 (1.69, 6.62)**	9.88 (4.22, 23.14)**	3.83 (1.88, 7.83)**	11.00 (4.43, 27.27)**
Sleep duration								
≥ 6 & < 9 h	**Reference**	**Reference**	**Reference**	**Reference**	**Reference**	**Reference**	**Reference**	**Reference**
< *6 h*	1.70 (1.15, 2.49)*	3.03 (1.85, 4.98)**	1.28 (0.81, 2.04)	2.00 (1.03, 3.88)*	1.26 (0.79, 2.00)	1.84 (0.93, 3.65)	0.88 (0.53, 1.44)	0.91 (0.42, 1.95)
≥ 9 h	1.51 (1.00, 2.28)*	1.56 (0.86, 2.82)	1.61 (0.98, 2.63)	1.32 (0.59, 2.95)	1.64 (1.00, 2.71)*	1.32 (0.58, 3.00)	1.97 (1.19, 3.28)*	1.73 (0.75, 3.98)

All models are constructed using multinomial logistic regression. The reference category was “robust”. Model 1: Adjusted for age, centre, *n* = 2393; Model 2: Adjusted for age, centre, BMI, pain, depression, alcohol and smoking, *n* = 1903; Model 3: Same as model 2 but additionally adjusted for total testosterone: *n* = 1885; Model 4: Same as model 3 but mutually adjusted for both sleep quality and sleep duration; *n* = 1885

^**^P < 0.001

^*^P < 0.05

## Discussion

In this observational study of middle aged and older European men, poor sleep quality was associated with higher levels of frailty. Duration of sleep less than 6 h and 9 or more hours was also weakly associated with frailty. Adjustment for level of testosterone did not influence the strength of these associations.

Our data are consistent with the majority (though not all) cross-sectional studies and a recent meta-analysis which suggests an association between sleep disturbance and frailty (Del Brutto, 2016 [8]; Nakakubo 2018 [12]; Ensrud 2009 [7]; Moreno-Tamayo, 2017 [43]; Lee WJ 2017 [16]; Ensrud 2012 [6]; Baniak 2018 [14]; Vaz Fragoso 2009 [44]; Endeshaw 2009 [9]; Sun 2020 [4]; Kang 2019 [15]; Pourmotabbed 2020 [45]). Sleep disturbance in these studies included both poor sleep quality, latency, efficiency and also daytime somnolence. Data from a prospective study in men suggests also that those with poor sleep quality are more likely to develop frailty [7]. Our data extend findings from these studies and show a dose response association with increasing sleep score associated with both an increased frailty index and an increased risk of being frail. In contrast to other studies we found a significant association also with prefrailty, though the magnitude of the association was weaker than for frailty.

Our data in relation to sleep duration and the frailty index are consistent also with the results of a meta-analysis where both the highest category (> 8 h) and lowest category (< 6 h) of sleep duration, when compared to the reference category (6–8 h), were significantly correlated with an increased risk of frailty [45]. In contrast though to subgroup analysis from the meta-analysis we observed a weak association between shorter sleep duration and an increased risk of prefrailty. More recently, a large prospective study (median follow up 4.4 years) of 7623 individuals demonstrated long, but not short sleep duration, was significantly associated with risk of incident frailty [46].

There are a number of possible mechanisms linking sleep and frailty. Sleep disorders may be a marker of conditions including poor health and co-morbidities which, by themselves, impair sleep and increase the likelihood of frailty or death [6]. Other possible mechanisms including hormonal factors have been suggested. We were interested in this analysis at the potential influence of testosterone on the relationship between sleep and frailty.

In our study we observed a small though non-significant decrease in total testosterone level with increasing sleep score, and slightly lower levels among those with shorter (< 6 h) or longer (9 or more hours). After adjustment for a range of confounders including BMI, compared to those with the lowest sleep score, those with the highest sleep score (15–20) were over twice as likely to have a low testosterone level (< 10.5ng/ml). To date most studies examining the relationship between sleep and testosterone have been interventional in design with few population data. In an analysis of data from MrOS, a population study of men aged 65 years and over, no association was found between duration of sleep and total testosterone levels, though lower levels were associated with increased nocturnal wakening’s and decreased sleep efficiency. Adjusting for BMI significantly attenuated the associations. Auyeung (2015) reported that total testosterone levels increased with sleep duration, from 5 h up until 9.9 h at which point the levels were found to decrease. This was true also for testosterone as measured either as free fraction or bioavailable fraction. Sleep latency and insomnia however were not found to be associated with testosterone levels. Using data from NHANES 2019 in a multivariate analysis among men aged 16 years and over serum testosterone appeared to decrease by 5.85 ng/dL per hour of sleep [22]. Recently, a USA based large retrospective cohort analysis of over 2 million men using electronic health records showed a higher rates of testosterone deficiency across three different sleep disorders; insomnia, sleep apnoea and circadian rhythm disturbance [23].

To our knowledge there are no data which have looked at whether any association between frailty and sleep can be explained by low testosterone. Our data showed that adjustment for testosterone did not influence the observed strength of association between sleep parameters and frailty. This suggests that testosterone is unlikely to be a major contributor to potential causal mechanisms linking the relationship between sleep and frailty.

Our study was population based and used standardised methods in assessment. There are however a number of limitations to be considered in interpreting the data. Firstly, the participants in this study are relatively young in comparison to other studies which may affect results obtained. We emphasise our data was cross-sectional in nature and it is not possible to determine the temporal nature of the association between sleep and frailty for which prospective data are needed. We did not utilise objective parameters such as actigraphy or polysomnography to assess sleep disorders. Instead, information about sleep was obtained by self-report and subject therefore to potential recall bias. Increasing frailty may influence perception of sleep quality for example. It should be noted that the sleep questionnaire used in this study, although shown to have good reliability and validity, is different to those used in most previous other studies [37]. In addition, a small proportion of men did not attend (due to death or had declined) for follow up assessment which was the point data on sleep parameters were collected. Of those that did attend follow up assessment, 12.5% (n = 343) were excluded due to incomplete data on either frailty measures or sleep parameters. Both these groups may have been more likely to be frailer/older which may impact on results. Finally our data were obtained in a predominately Caucasian group and the results should not be generalised beyond this [47].

In conclusion, frailty is associated with impaired sleep quality, and also duration of sleep. The association cannot, however, be explained by variation in testosterone levels.

### Supplementary Information

**Additional file 1.** 

## Data Availability

All data generated or analysed during this study are included in this published article and supplementary files.

## Abbreviations

FI: Frailty Index
CI: Confidence Interval
BDI: Becks Depression Inventory
BMI: Body Mass Index
EMAS: European Male Ageing Study
BDI: Becks Depression Inventory
T: Testosterone
