# Supplementary material for: Does testosterone influence the association between sleep and frailty in men: results from the European Male Aging Study
Source: BMC Geriatr. 2023 Dec 6;23:813. doi: 10.1186/s12877-023-04450-8 (PMC10702097; doi:10.1186/s12877-023-04450-8)
Supplement: Supplementary file 1 — Additional file 1. [file 12877_2023_4450_MOESM1_ESM.docx]

Supplementary Material

Supplementary Table 1: Variables included in the EMAS frailty index

| **Origin** | **Variable** | **Cut Point** |
| --- | --- | --- |
|  |  |  |
| SF-36 | Rating general health |  |
| SF-36 activities of daily living | Help feeding yourself | Limited or limited a little = 1, not limited = 0 |
|  | Help walking in your home | Limited or limited a little = 1, not limited = 0 |
|  | Help bathing and dressing yourself | Limited or limited a little = 1, not limited = 0 |
|  | Walking 1 km | Limited =1, limited a little = 0.5, not limited = 0 |
|  | Walking more than 1 km | Limited =1, limited a little = 0.5, not limited = 0 |
|  | Climbing one flight of stairs | Limited =1, limited a little = 0.5, not limited = 0 |
|  | Climbing several flights of stairs | Limited =1, limited a little = 0.5, not limited = 0 |
|  | Unable to do moderate activity | Limited =1, limited a little = 0.5, not limited = 0 |
|  | Unable to do vigorous activity | Limited =1, limited a little = 0.5, not limited = 0 |
| During the past 4 weeks have you had any of the following problems | |  |
| SF-36 | Accomplish less than you would like as  a result of your physical health | All or most of time = 1, sometimes = 0.5, little time or none = 0 |
| SF-36 | Cut down on the amount of time spent on work or other activities as a result of emotional problems | All or most of time = 1, sometimes = 0.5, little time or none = 0 |
| Questions on how you feel and how things have been with you during the past 4 weeks | | |
| SF-36 | Full of life | Little time or none = 1, sometimes = 0.5, all or most of time = 0 |
|  | In the dumps | All or most of time = 1, sometimes = 0.5, little time or none = 0 |
|  | Down hearted | All or most of time = 1, sometimes = 0.5, little time or none = 0 |
|  | Tired | All or most of time = 1, sometimes = 0.5, little time or none = 0 |
| SF-36: During the past 6 months have you experienced serious illness or injury | | Yes = 1, no = 0 |
| Beck’s Depression Inventory | Change in sleep pattern | Lot less or lot more = 1, same or little change = 0 |
|  | Concentration difficulty | All or most of time = 1, fair = 0.5, OK = 0 |
| International Prostate Symptom Score | Over the past month, how often have you had to postpone urination | Always / > 50% = 1, about or less than 50% = 0.5, not at all / < 20% = 0 |
|  | Night urinate | 2 times or more = 1, 0 or 1 = 0 |
|  | Weak stream | Always / > 50% = 1, about or less than 50% = 0.5, not at all / < 20% = 0 |
| Self-reported morbidities |  |  |
|  | Heart condition | Yes = 1, no = 0 |
|  | High blood pressure | Yes = 1, no = 0 |
|  | Bronchitis | Yes = 1, no = 0 |
|  | Asthma | Yes = 1, no = 0 |
|  | Diabetes | Yes = 1, no = 0 |
|  | Liver condition | Yes = 1, no = 0 |
|  | Kidney condition | Yes = 1, no = 0 |
|  | Prostate disease | Yes = 1, no = 0 |
|  | Thyroid disease | Yes = 1, no = 0 |
|  | Cancer (ever) | Yes = 1, no = 0 |
|  | Stroke (ever) | Yes = 1, no = 0 |
| Continuous variable cut points – highest or lowest 10^th^ centile | | *Cut off point corresponds to the lowest (worst performing) 10^th^ centile* |
| Cognition | Copying ROCF | Score < 28 = 1, ≥ 28 = 0 |
|  | Delayed reproduction ROCF | Score < 8 = 1, ≥ 8 = 0 |
|  | Camden Topographical Recognition Memory | Score < 16 = 1, ≥ 16 = 0 |
|  | Digit-Symbol Substitution test | Score < 16 = 1, ≥ 16 = 0 |
| Tinetti |  | Score < 25 = 1, ≥ 25 = 0 |
|  |  | *Cut off point corresponds to the highest (worst performing) 10^th^ centile* |
| Physical Performance test | Time to walk 15.4 m | Time ≥ 16.7s = 1, < 16.7s = 0 |
|  |  |  |

# Supplementary Table 2. Sleep Questionnaire used in EMAS study (adapted from Jenkins sleep questionnaire, Jenkins, Stanton et al. 1988)

# Supplementary Table 3: Association between free testosterone, sleep quality score and sleep duration

| Characteristics | Free Testosterone (pmol/L) | β [nmol/L change in free testosterone] (95% CI) | | Low free Testosterone (<220 pmol/L) | Odds ratio for low free testosterone (95% CI) | |
| --- | --- | --- | --- | --- | --- | --- |
|  | *Mean (SD)* | *Model 1*^1^ | *Model 2*^2^ | *N (%)* | *Model 3*^3^ | *Model 4*^4^ |
| *Sleep quality category* |  |  |  |  |  |  |
| 0-4 | 289.9 (92.1) | **Reference** | **Reference** | 277 (20.72) | **Reference** | **Reference** |
| 5-9 | 278.9 (90.3) | -4.7 (-13.06, 3.65) | -6.22 (-15.52, 3.09) | 142 (22.33) | 0.99 (0.78, 1.27) | 0.95 (0.72, 1.26) |
| 10-14 | 281.9 (100.1) | -5.82 (-17.7, 6.09) | -1.74 (-15.41, 11.93) | 55 (22.09) | 1.07 (0.76, 1.51) | 0.96 (0.63, 1.46) |
| 15-20 | 254.9 (121.0) | -16.13 (-34.5, 2.21) | -20.02 (-40.90, 0.87) | 31 (32.63) | 1.35 (0.84, 2.18) | 1.57 (0.87, 2.82) |
| *Sleep duration* |  |  |  |  |  |  |
| ≥6 & <9 hours | 287.9 (94.5) | **Reference** | **Reference** | 418 (21.23) | **Reference** | **Reference** |
| *<6 hours* | 268.04 (90.0) | -7.65 (-20.55, 5.26) | -6.12 (-20.35, 8.12) | 47 (23.98) | 0.92 (0.64, 1.33) | 0.87 (0.58, 1.33) |
| ≥9 hours | 263.6 (92.2) | -11.89 (-26.40, 2.62) | -13.28 (-29.10, 2.54) | 40 (26.32) | 1.10 (0.74, 1.63) | 1.05 (0.66, 1.66) |

Models 1 and 2 are constructed using linear regression. Models 3 and 4 are constructed using logistic regression. Model 1: Adjusted for age and centre, n=2351; Model 2: adjusted for age, centre, BMI, depression, pain, smoking status and alcohol intake, n=1885; Model 3: Adjusted for age and centre, n=2351; Model 4: Adjusted for age, centre, BMI, depression, pain, smoking status and alcohol intake, n=1885

*p<0.05

# Supplementary Table 4: Association between sleep quality, sleep duration and frailty index with adjustment for free testosterone

| Sleep Variable | Model 1 | Model 2 | Model 3 | Model 4 |
| --- | --- | --- | --- | --- |
|  | *Relative frailty index score (95% CI)* | | | |
| *Sleep quality category* |  |  |  |  |
| 0-4 | **Reference** | **Reference** | **Reference** | **Reference** |
| 5-9 | 1.40 (1.32, 1.49)** | 1.26 (1.18, 1.34)** | 1.27 (1.19, 1.34)** | 1.27 (1.20, 1.35)** |
| 10-14 | 1.80 (1.65, 1.96)** | 1.37 (1.25, 1.49)** | 1.37 (1.26, 1.50)** | 1.39 *1.27, 1.51)** |
| 15-20 | 2.47 (2.17, 2.81)** | 1.57 (1.38, 1.78)** | 1.49 (1.31, 1.70)** | 1.51 (1.32, 1.73)** |
| *Sleep duration* |  |  |  |  |
| ≥6 & <9 hours | **Reference** | **Reference** | **Reference** | **Reference** |
| *<6 hours* | 1.43 (1.30, 1.58)** | 1.16 (1.06, 1.28)** | 1.14 (1.04, 1.25)* | 1.02 (0.92, 1.12) |
| ≥9 hours | 1.13 (1.01, 1.27)* | 1.11 (1.00, 1.23)* | 1.13 (1.01, 1.25)* | 1.19 (1.07, 1.32)* |

Table to show negative binomial regression analysis of sleep quality and sleep duration against frailty index. Results are reported as IRRs with 95% confidence intervals (CIs) **P<0.001 *P<0.05) Model 1: Sleep quality against frailty, adjusted for age, centre; and Sleep duration against frailty, adjusted for age, centre: n=2393. Model 2: Sleep quality against frailty, adjusted for age, centre, BMI, pain, depression, alcohol and smoking; and Sleep duration against frailty, adjusted for age, centre, BMI, pain, depression, alcohol and smoking n=2351. Model 3: Same as model 2 but additionally adjusted for ***free testosterone***: n=1885. Model 4: Same as model 3 but contains both sleep quality and sleep duration; n=1885.

**P<0.001

*P<0.05)

# Supplementary Table 5: Association between sleep quality, sleep duration and frailty categories with adjustment for free testosterone

| Sleep Variable | Model 1 |  | Model 2 |  | Model 3 |  | Model 4 |  |
| --- | --- | --- | --- | --- | --- | --- | --- | --- |
|  | **Pre-frail** | **Frail** | **Pre-frail** | **Frail** | **Pre-frail** | **Frail** | **Pre-frail** | **Frail** |
| *Sleep quality* |  |  |  |  |  |  |  |  |
| 0-4 | **Reference** | **Reference** | **Reference** | **Reference** | **Reference** | **Reference** | **Reference** | **Reference** |
| 5-9 | 2.30 (1.76, 3.03)** | 2.93 (1.88, 4.56)** | 1.89 (1.37, 2.61)** | 1.91 (1.09, 3.34)* | 1.93 (1.39, 2.67)** | 2.01 (1.14, 3.53)* | 2.01 (1.44, 2.79)** | 2.08 (1.18, 3.66)* |
| 10-14 | 4.17 (2.91, 5.99)** | 6.82 (3.97, 11.72)** | 2.58 (1.66, 4.02)** | 2.72 (1.35, 5.48)* | 2.66 (1.70, 4.17)** | 2.75 (1.34, 5.63)* | 2.93 (1.84, 4.66)** | 2.99 (1.44, 6.23)* |
| 15-20 | 7.89 (4.41, 14.11)** | 41.90 (21.93, 80.03)** | 3.39 (1.71, 6.71)** | 10.93 (4.76, 25.14)** | 3.13 (1.56, 6.26)* | 9.29 (1.10, 1.22)** | 3.59 (1.18, 3.28)* | 1.55 (4.22, 26.36)** |
| *Sleep duration* |  |  |  |  |  |  |  |  |
| ≥6 & <9 hours | **Reference** | **Reference** | **Reference** | **Reference** | **Reference** | **Reference** | **Reference** | **Reference** |
| *<6 hours* | 1.70 (1.15, 2.49)* | 3.03 (1.85, 4.98)** | 1.28 (0.81, 2.04) | 2.00 (1.03, 3.88)* | 1.25 (0.78, 1.99) | 1.75 (0.88,3.48) | 0.88 (0.53, 1.44) | 0.87 (0.40, 1.86) |
| ≥9 hours | 1.51 (1.00, 2.28)* | 1.56 (0.86, 2.82) | 1.61 (0.98, 2.63) | 1.32 (0.59, 2.95) | 1.65 (1.00, 2.72)* | 1.33 (.59, 3.01) | 1.97 (1.18, 3.28)* | 1.73 (1.10, 1.22) |

Table to show multinomial logistic regression analysis of sleep quality and sleep duration against frailty index, compared to reference group “robust”. Results are reported as relative rate ratios (RRRs) with 95% confidence intervals (CIs) **P<0.001 *P<0.05) Model 1: Sleep quality against frailty, adjusted for age, centre; and Sleep duration against frailty, adjusted for age, centre: n=2393. Model 2: Sleep quality against frailty, adjusted for age, centre, BMI, pain, depression, alcohol and smoking; and Sleep duration against frailty, adjusted for age, centre, BMI, pain, depression, alcohol and smoking n=2351. Model 3: Same as model 2 but additionally adjusted for ***free testosterone***: n=1885. Model 4: Same as model 3 but contains both sleep quality and sleep duration; n=1885
